# Emergency Surgery Independently Predicts Postoperative Recurrence in Patients with Pathologic T3N0M0 Colon Cancer: A Retrospective Single-Center Cohort Study

**DOI:** 10.3390/curroncol33090526

**Published:** 2026-09-01

**Authors:** Selahattin Çelik, Salih Karatlı, Esra Zeynelgil, Hatice Ayyıldız Sevim, Tülay Eren

**Affiliations:** Department of Medical Oncology, Ankara Etlik City Hospital, 06010 Ankara, Türkiye

**Keywords:** colon cancer, pathologic T3N0M0, stage II colon cancer, emergency surgery, recurrence, prognostic factors, disease-free survival, risk stratification

## Abstract

Background and Objectives: Pathologic stage IIA (T3N0M0) colon cancer represents a heterogeneous subgroup of stage II disease with variable recurrence risk despite similar TNM staging. Although several clinicopathological high-risk features are incorporated into current treatment guidelines, the independent prognostic significance of emergency surgery remains incompletely understood. This study aimed to identify clinicopathological predictors of postoperative recurrence, with particular emphasis on the prognostic impact of emergency surgery. Materials and Methods: This retrospective single-center cohort study included 118 patients with pathologic T3N0M0 colon adenocarcinoma who underwent curative-intent surgery. Demographic, clinicopathological, treatment, and follow-up data were analyzed. Univariable and multivariable logistic regression analyses were performed to identify independent predictors of recurrence. Because adjuvant chemotherapy was not randomly assigned, a sensitivity analysis including adjuvant chemotherapy was conducted to address confounding by indication. Disease-free survival (DFS) was evaluated using the Kaplan–Meier method. Results: During follow-up, recurrence occurred in 15 patients (12.7%). Patients with recurrence underwent emergency surgery significantly more frequently than those without recurrence (60.0% vs. 20.4%, *p* = 0.003). Perineural invasion and adjuvant chemotherapy were associated with recurrence in univariable analyses. In the primary multivariable model, emergency surgery remained an independent predictor of recurrence (OR 5.508, 95% CI 1.726–17.576; *p* = 0.004), whereas perineural invasion showed only borderline significance. In the sensitivity analysis, emergency surgery remained independently associated with recurrence (OR 4.079, 95% CI 1.207–13.779; *p* = 0.024), while adjuvant chemotherapy was no longer independently associated with recurrence. Kaplan–Meier analysis demonstrated significantly shorter DFS among patients undergoing emergency surgery (log-rank *p* = 0.002). Conclusions: Among patients with pathologic T3N0M0 colon cancer, the need for emergency surgery was associated with a higher risk of recurrence after adjustment for other clinicopathological factors. Emergency presentation may therefore serve as an additional marker for identifying patients at increased postoperative risk. Further prospective multicenter research is needed to confirm this association and to explore its potential value when combined with molecular biomarkers for individualized postoperative management.

## Simple Summary

Colon cancer can return after apparently successful surgery, even in patients whose cancer has not spread to the lymph nodes or distant organs. Identifying patients at higher risk of recurrence is important for planning follow-up and postoperative treatment. In this study, we evaluated 118 patients with a specific form of stage II colon cancer who underwent surgery with the intention of cure. Cancer recurrence occurred in 15 patients (12.7%). Patients who required emergency surgery because of acute tumor-related complications were more likely to experience recurrence than those who underwent planned surgery. After taking other clinical and pathological factors into account, emergency surgery remained independently associated with an increased risk of recurrence. Patients who underwent emergency surgery also had shorter disease-free survival. These findings suggest that the circumstances in which surgery is performed may provide additional information about the risk of cancer recurrence. Patients requiring emergency surgery may therefore benefit from closer postoperative monitoring and individualized assessment. Larger prospective studies are needed to confirm these findings and determine how they can best be incorporated into clinical practice.

## 1. Introduction

Colorectal cancer (CRC) remains one of the most frequently diagnosed malignancies and a leading cause of cancer-related mortality worldwide. According to GLOBOCAN 2022 estimates, more than 1.9 million new CRC cases and approximately 904,000 related deaths occurred globally. Despite improvements in screening, surgical techniques, pathological assessment, and systemic treatments, postoperative recurrence continues to limit long-term survival after curative-intent [1].

Complete oncological resection with adequate regional lymphadenectomy is the cornerstone of treatment for localized colon cancer. Pathological tumor-node-metastasis staging remains the principal determinant of prognosis and postoperative treatment; however, patients within the same pathological stage may have markedly different recurrence risks. This variability is particularly relevant in stage II colon cancer, which represents a biologically and clinically heterogeneous disease group [2,3].

Most patients with stage II colon cancer are cured by surgery alone, whereas a clinically relevant subgroup develops recurrence. Consequently, a major challenge in postoperative management is to identify patients whose recurrence risk is sufficiently high to justify adjuvant chemotherapy while avoiding unnecessary treatment-related toxicity [2,3,4].

Pathological T3N0M0 disease, corresponding to stage IIA colon cancer, constitutes an important subgroup within stage II disease. Although T3N0M0 disease generally has a more favorable prognosis than T4 or node-positive disease, recurrence still occurs in a meaningful minority of patients. Because T4 status—the strongest conventional risk factor within stage II disease—is absent in this population, other clinical and histopathological characteristics may assume greater prognostic importance. Evaluating a cohort restricted to pathologic T3N0M0 disease may therefore help clarify the prognostic contribution of individual risk factors that can be obscured when T3 and T4 tumors are analyzed together [2,3].

Routine adjuvant chemotherapy is not recommended for patients with stage II colon cancer who lack high-risk features. Current guidelines recommend individualized decision-making based on recurrence risk, molecular characteristics, comorbidities, and patient preferences. ASCO identifies T4 disease as an indication for adjuvant therapy and recognizes fewer than 12 examined lymph nodes, lymphovascular or perineural invasion, bowel obstruction, tumor perforation, poorly or undifferentiated histology, and grade BD3 tumor budding as additional adverse features [2]. ESMO similarly emphasizes clinicopathological risk stratification when considering adjuvant treatment in stage II disease [3].

Nevertheless, currently accepted high-risk characteristics do not have equivalent prognostic effects and may reflect either aggressive tumor biology or the circumstances of surgery and pathological assessment. Furthermore, these features frequently coexist, making it difficult to determine whether an individual factor independently predicts recurrence. This contributes to continuing uncertainty regarding which patients with T3N0M0 disease truly benefit from adjuvant chemotherapy [2,5].

Perineural invasion and lymphovascular invasion are well-established markers of tumor dissemination associated with adverse outcomes in node-negative colorectal cancer. Studies restricted to stage II disease have reported inferior disease-free and overall survival among patients with perineural invasion, although its independent effect may be attenuated after adjustment for other pathological variables [6,7,8,9]. Similarly, lymphovascular invasion has been associated with an increased risk of recurrence and mortality, supporting its inclusion among recognized high-risk features [10,11].

Accurate nodal assessment is a key component of reliable pathological staging. When fewer than 12 lymph nodes are examined, occult nodal involvement may be missed, potentially resulting in stage misclassification. The number of lymph nodes retrieved is not determined solely by the surgical procedure but may vary according to pathological examination, tumor site, patient-related characteristics, and the circumstances under which surgery is performed. Consequently, inadequate nodal evaluation may function both as a marker of potentially incomplete staging and as a surrogate for variation in surgical or pathological quality [2,3].

Tumor budding is another relevant histopathological marker in stage II colon cancer. Standardized assessment criteria were established by the International Tumor Budding Consensus Conference, and prospective findings from the SACURA trial further demonstrated its prognostic relevance. High-grade BD3 budding is now included among the adverse features considered in adjuvant treatment decisions [2,12,13].

Molecular characteristics further contribute to the heterogeneity of stage II disease. Deficient mismatch repair and microsatellite instability-high tumors generally have a more favorable stage-adjusted prognosis and derive limited benefit from fluoropyrimidine monotherapy in the absence of other compelling indications. More recently, postoperative circulating tumor DNA has emerged as a highly promising marker of molecular residual disease. In the randomized DYNAMIC trial, a ctDNA-guided strategy reduced the use of adjuvant chemotherapy without compromising recurrence-free survival, and subsequent five-year follow-up continued to support the prognostic and treatment-guiding potential of postoperative ctDNA [14,15]. Nevertheless, ctDNA testing is not yet universally available, and conventional clinicopathological variables remain the principal basis of postoperative risk assessment in many healthcare settings.

Among these readily available variables, emergency surgery deserves particular attention. Emergency operations are typically required because of acute tumor-related complications such as bowel obstruction, perforation, or bleeding. Emergency presentation may reflect a complex interaction between tumor biology, patient characteristics, and healthcare-related factors and has been associated with adverse clinicopathological features [16].

Emergency colorectal cancer surgery is consistently associated with greater perioperative morbidity and mortality than elective surgery. Recent meta-analyses and population-based studies have also reported inferior recurrence-free and overall survival following emergency resection [17,18]. However, the interpretation of this association is challenging because emergency surgery is closely linked to obstruction, perforation, advanced T stage, inadequate preoperative staging, postoperative complications, and reduced lymph-node yield. Whether emergency surgery independently predicts recurrence after adjustment for these factors therefore remains uncertain, particularly in patients specifically restricted to pathologic T3N0M0 [16,18].

Therefore, the present study aimed to identify clinicopathological factors associated with recurrence in patients with pathologic T3N0M0 colon cancer following curative-intent surgery, with particular emphasis on evaluating whether emergency surgery independently predicts postoperative recurrence after adjustment for established prognostic factors.

## 2. Materials and Methods

### 2.1. Study Design and Patient Population

We retrospectively evaluated consecutive patients with histopathologically confirmed T3N0M0 colon adenocarcinoma who had undergone curative-intent surgery at Ankara Etlik City Hospital. Eligible patients underwent surgery between 1 January 2018 and 7 April 2026. To ensure that all study procedures preceded Institutional Review Board approval, eligibility was restricted to patients diagnosed and treated before 8 April 2026, the date on which ethical approval was granted. Relevant clinical, pathological, treatment, and follow-up information was obtained retrospectively from institutional electronic health records, pathology and operative reports, and medical oncology databases.

Eligibility required an age of ≥18 years, histologically confirmed colon adenocarcinoma, curative-intent surgical resection, and a postoperative pathological stage of T3N0M0 according to the eighth edition of the American Joint Committee on Cancer (AJCC) TNM classification. Inclusion in the analysis additionally required complete clinicopathological information and sufficient follow-up data.

Patients were excluded if they had rectal cancer, pathological T1, T2, or T4 disease, regional lymph-node metastasis, distant metastatic disease at diagnosis, positive surgical margins (R1/R2), synchronous malignancy, non-adenocarcinoma histology, received neoadjuvant chemotherapy or radiotherapy, died within 30 days after surgery, or had incomplete clinical or follow-up data.

A total of 118 patients fulfilled the eligibility criteria and were included in the final analysis.

### 2.2. Clinicopathological Assessment

Pathological staging was based on the eighth edition of the American Joint Committee on Cancer (AJCC) TNM classification. Tumors were classified as T3 when they extended beyond the muscularis propria into the surrounding pericolorectal tissues but did not penetrate the visceral peritoneal surface or directly involve neighboring organs. N0 disease indicated that no metastases were identified in the regional lymph nodes.

Histopathological evaluations were performed by experienced gastrointestinal pathologists according to routine institutional practice. The recorded pathological variables included tumor location, histological differentiation, lymphovascular invasion (LVI), perineural invasion (PNI), tumor perforation, number of examined lymph nodes, and resection margin status.

Adequate lymph-node evaluation was defined as examination of at least 12 lymph nodes, whereas examination of fewer than 12 lymph nodes was considered inadequate according to current international recommendations.

Tumor differentiation was categorized as well differentiated, moderately differentiated, poorly differentiated, or unknown based on the original pathology reports.

### 2.3. Clinical Variables

Patient-related variables collected for the analysis comprised age at diagnosis, sex, smoking history, Eastern Cooperative Oncology Group (ECOG) performance status, Charlson Comorbidity Index (CCI), American Society of Anesthesiologists (ASA) score, body mass index (BMI), and laboratory measurements obtained at baseline.

For analysis, primary tumors were grouped by anatomical site. Right-sided tumors included those arising in the cecum, ascending colon, hepatic flexure, or transverse colon, whereas tumors of the splenic flexure, descending colon, or sigmoid colon were classified as left-sided.

Preoperative serum levels of carcinoembryonic antigen (CEA) and carbohydrate antigen 19-9 (CA19-9) were extracted from the available clinical records.

Emergency surgery was defined as an unplanned urgent operation performed because of an acute tumor-related clinical presentation that precluded planned elective management, based on the documented clinical and surgical records. The exact interval from symptom onset or hospital admission to surgery was not systematically recorded; therefore, no predefined time-based threshold was applied. Specific indications for emergency surgery were not systematically recorded as mutually exclusive categories, while perforation was recorded separately as a clinicopathological variable.

### 2.4. Adjuvant Treatment

Postoperative adjuvant chemotherapy was administered according to the contemporary international guidelines applicable during the study period and the decision of the institutional multidisciplinary tumor board.

Treatment selection was individualized according to patient age, performance status, comorbidities, postoperative recovery, pathological risk factors, and physician judgment.

Patients received either oxaliplatin-based combination chemotherapy (FOLFOX or CAPOX), fluoropyrimidine monotherapy (capecitabine), or no adjuvant chemotherapy. Adjuvant treatment was administered for 3–6 months, depending on the selected regimen, pathological risk profile, treatment tolerance, and prevailing guideline recommendations.

For statistical analyses, patients were evaluated both according to specific chemotherapy regimen and according to receipt of any adjuvant chemotherapy.

### 2.5. Follow-Up and Outcome Measures

Following curative-intent surgery, patients were routinely followed in accordance with institutional practice and contemporary international guidelines. Follow-up evaluations included physical examination, laboratory investigations including serum CEA measurement, colonoscopic surveillance, and thoracoabdominal imaging at clinically appropriate intervals.

The primary endpoint of the study was tumor recurrence.

A recurrence event was considered to have occurred when local, regional, or distant disease was documented by radiological, histopathological, or clinical assessment after curative-intent resection.

Disease-free survival (DFS) was calculated from the date of curative surgery to the earliest occurrence of either documented disease recurrence or death from any cause. For patients who remained alive and recurrence-free, follow-up was censored on the date of their most recent assessment.

### 2.6. Statistical Analysis

Statistical analyses were conducted with IBM SPSS Statistics, version 25.0 (IBM Corp., Armonk, NY, USA).

The distribution of continuous variables was assessed by visual inspection and the Shapiro–Wilk test. Data showing a normal distribution were summarized as mean ± standard deviation (SD), while variables with a non-normal distribution were reported as median (minimum–maximum). Categorical data were described using numbers and percentages.

For comparisons between two independent groups, the independent-samples t-test was applied to normally distributed continuous variables and the Mann–Whitney U test to non-normally distributed variables, as appropriate. Associations between categorical variables were examined using Pearson’s chi-square test or Fisher’s exact test.

Potential predictors of recurrence were first examined using univariable binary logistic regression. Variables yielding a *p*-value < 0.10 in these analyses, along with variables considered clinically relevant, were subsequently considered for the multivariable logistic regression model. Effect estimates are reported as odds ratios (ORs) with 95% confidence intervals (CIs).

Because adjuvant chemotherapy was administered according to clinical indications rather than random allocation, it was not included in the primary multivariable model to minimize confounding by indication. A sensitivity multivariable analysis including adjuvant chemotherapy was subsequently performed to evaluate the robustness of the findings.

Disease-free survival was estimated using the Kaplan–Meier method and compared using the log-rank test. Median survival times with corresponding 95% confidence intervals were reported where appropriate.

All statistical tests were two-sided, and a *p*-value < 0.05 was considered statistically significant.

### 2.7. Ethical Approval

The study was conducted in accordance with the ethical principles of the Declaration of Helsinki and was approved by the Clinical Research Ethics Committee of Ankara Etlik City Hospital (approval number AEŞH-BADEK1-2026-363; approval date: 8 April 2026).

Given the retrospective observational design of the study and the use of anonymized routinely collected clinical data, the requirement for written informed consent was waived by the Institutional Review Board.

## 3. Results

### 3.1. Baseline Characteristics

A total of 118 patients with pathologically confirmed T3N0M0 colon adenocarcinoma were included in the study. The median age was 64 years (range, 27–89 years), and 77 patients (65.3%) were male. Emergency surgery was performed in 30 patients (25.4%). Tumor-related perforation was identified in 6 patients (5.1%), whereas inadequate lymph node evaluation (<12 examined lymph nodes) was observed in 19 patients (16.1%). Perineural invasion and lymphovascular invasion were present in 23 (19.5%) and 31 patients (26.3%), respectively. Overall, 78 patients (66.1%) received adjuvant chemotherapy, and disease recurrence occurred in 15 patients (12.7%) during follow-up. The baseline demographic, clinical, and pathological characteristics of the study population are summarized in Table 1.

### 3.2. Comparison According to Adjuvant Chemotherapy

Emergency surgery was substantially more common among patients treated with adjuvant chemotherapy than among those who did not receive adjuvant treatment (37.2% vs. 2.5%, *p* < 0.001). The adjuvant chemotherapy group also had higher rates of perineural invasion (29.5% vs. 0%, *p* < 0.001) and lymphovascular invasion (39.7% vs. 0%, *p* < 0.001).

The two groups did not differ significantly in terms of age, sex, comorbidity status, tumor location, tumor perforation, adequacy of lymph-node evaluation, histological differentiation, microsatellite status, or baseline serum CEA and CA19-9 levels.

Recurrence was observed in 17.9% of patients who received adjuvant chemotherapy compared with 2.5% of those who did not (*p* = 0.019). A detailed comparison of demographic, clinical, and pathological characteristics according to adjuvant chemotherapy exposure is provided in Table 2.

### 3.3. Comparison According to Recurrence

Patients who developed recurrence underwent emergency surgery significantly more frequently than those without recurrence (60.0% vs. 20.4%, *p* = 0.003). Median baseline serum CEA levels were also significantly higher in patients who experienced recurrence (*p* = 0.005).

Perineural invasion was observed in 40.0% of patients who experienced recurrence and in 16.5% of those who remained recurrence-free; however, this difference was not statistically significant (*p* = 0.073). No significant between-group differences were identified for age, sex, comorbidity status, tumor location, tumor perforation, adequacy of lymph-node examination, lymphovascular invasion, histological differentiation, microsatellite status, or baseline serum CA19-9 levels.

Detailed comparisons between patients with and without recurrence are shown in Table 3.

### 3.4. Logistic Regression Analysis

Univariable logistic regression analysis identified emergency surgery (OR, 5.857; 95% CI, 1.875–18.292; *p* = 0.002), perineural invasion (OR, 3.373; 95% CI, 1.061–10.723; *p* = 0.039), and receipt of adjuvant chemotherapy (OR, 8.531; 95% CI, 1.079–67.437; *p* = 0.042) as significant predictors of recurrence.

No significant association with recurrence was found for male sex, age, comorbidity status, tumor location, baseline serum CEA or CA19-9 levels, lymph-node adequacy, lymphovascular invasion, or microsatellite status.

Variables meeting the *p* < 0.10 threshold in the univariable analysis were subsequently included in the primary multivariable logistic regression model. Adjuvant chemotherapy was intentionally excluded from this model because treatment allocation was non-randomized and patients with unfavorable clinicopathological characteristics were substantially more likely to receive postoperative chemotherapy, introducing the possibility of confounding by indication.

After multivariable adjustment, emergency surgery continued to show an independent association with recurrence (adjusted OR, 5.508; 95% CI, 1.726–17.576; *p* = 0.004). In contrast, the association between perineural invasion and recurrence did not reach statistical significance (adjusted OR, 3.021; 95% CI, 0.886–10.300; *p* = 0.077).

The results of the univariable and multivariable logistic regression analyses are summarized in Table 4.

### 3.5. Sensitivity Analysis

To evaluate the robustness of the primary model, a sensitivity multivariable logistic regression analysis including adjuvant chemotherapy was performed.

Emergency surgery remained an independent predictor of recurrence (adjusted OR, 4.079; 95% CI, 1.207–13.779; *p* = 0.024). In contrast, neither adjuvant chemotherapy (adjusted OR, 3.515; 95% CI, 0.377–32.768; *p* = 0.270) nor perineural invasion (adjusted OR, 2.324; 95% CI, 0.662–8.161; *p* = 0.188) was independently associated with recurrence after adjustment.

These findings are presented in Table 5.

### 3.6. Survival Analysis

**Figure 1:**
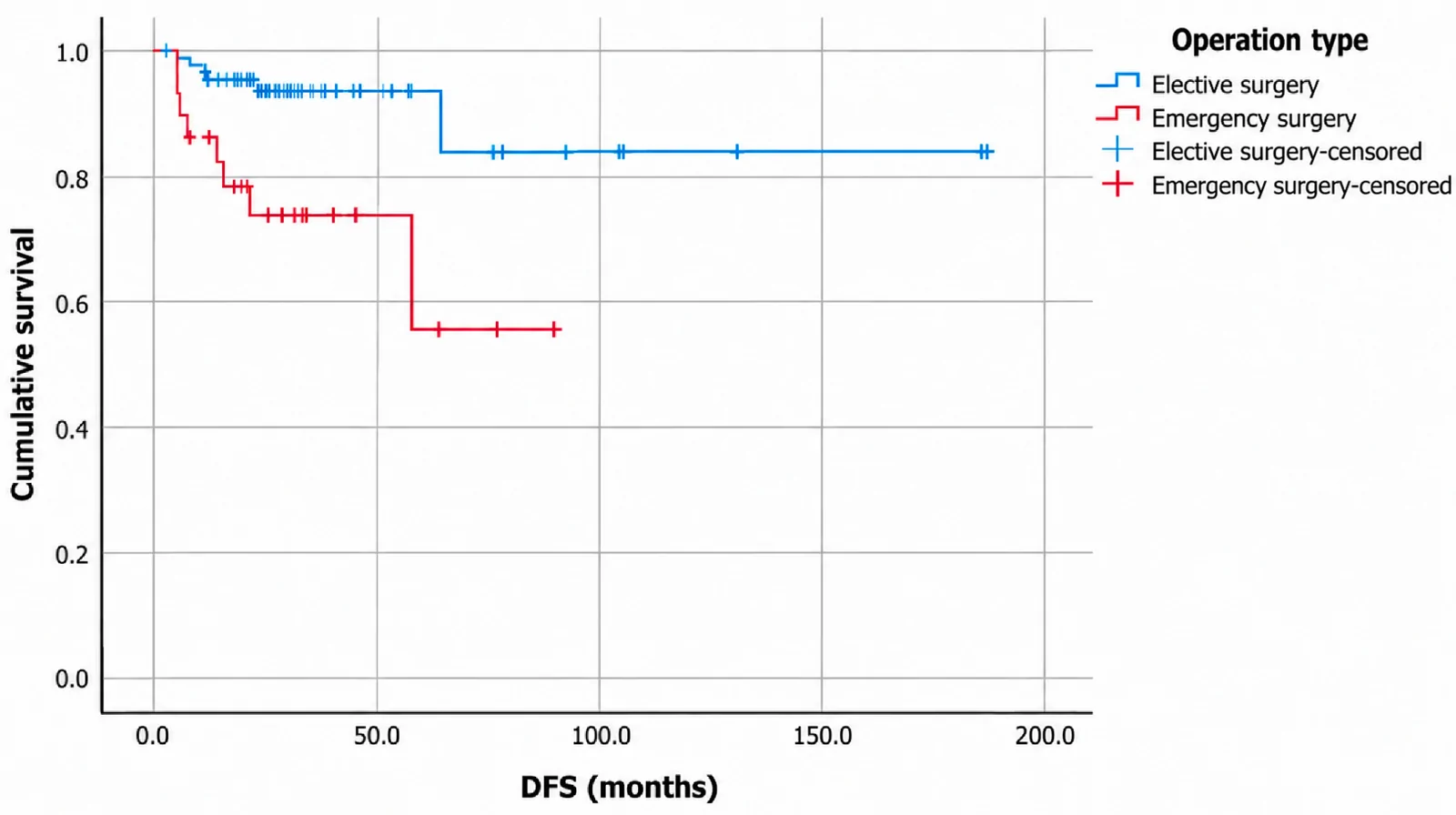
Kaplan–Meier analysis of disease-free survival according to type of surgery. Patients who underwent emergency surgery had significantly shorter disease-free survival than those who underwent elective surgery (log-rank *p* = 0.002). Abbreviation: DFS, disease-free survival.

Kaplan–Meier analysis demonstrated significantly shorter disease-free survival among patients undergoing emergency surgery than among those who underwent elective surgery (log-rank χ^2^ = 9.328, *p* = 0.002). Although the median disease-free survival was not reached in either group, the survival distributions differed significantly. Visual inspection of the Kaplan–Meier curves suggested that separation between the emergency and elective surgery groups occurred relatively early during follow-up and persisted thereafter (Figure 1).

**Figure 2:**
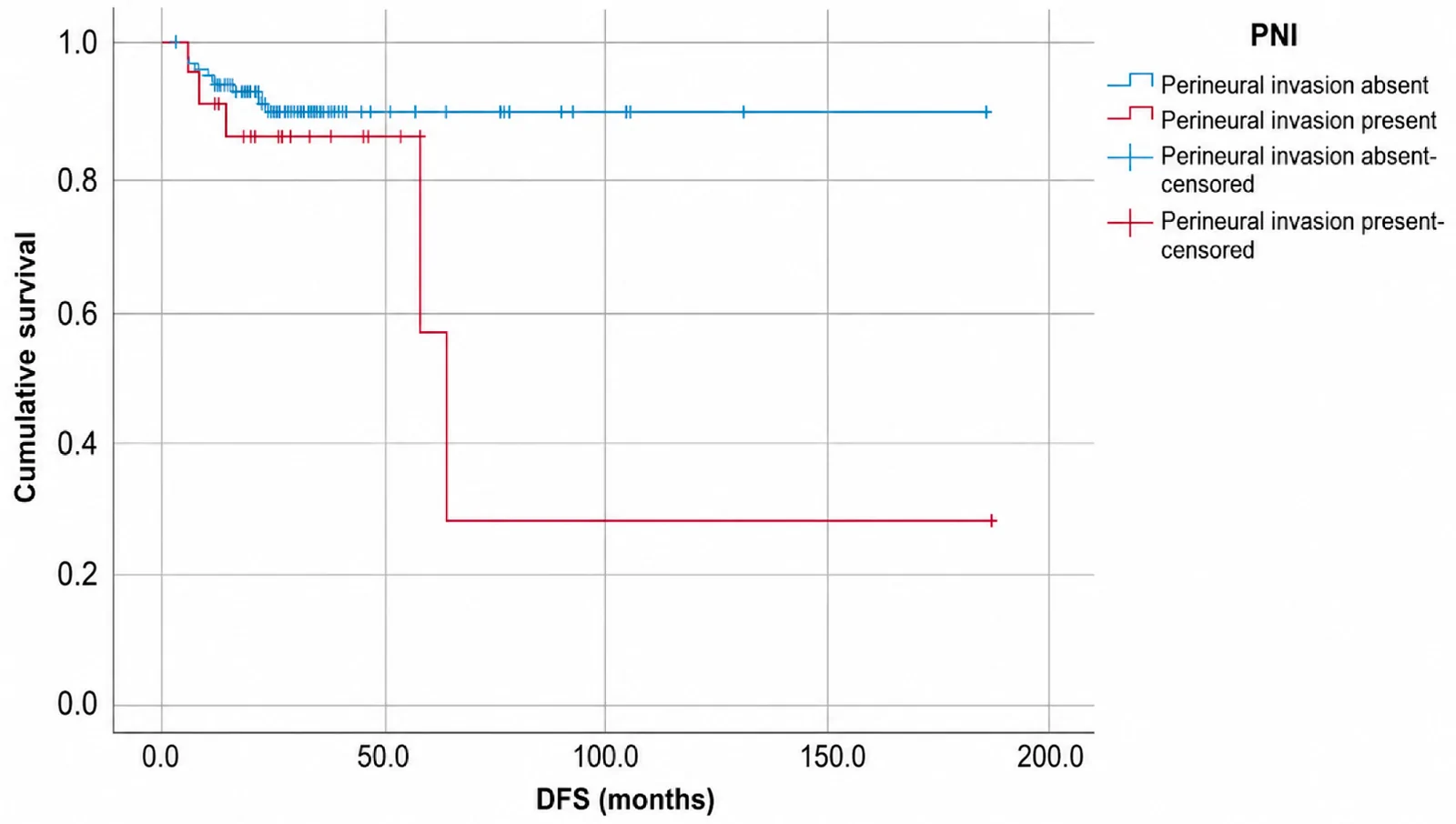
Kaplan–Meier analysis of disease-free survival according to perineural invasion status. Patients with perineural invasion showed less favorable disease-free survival than those without perineural invasion; however, the difference between the groups was not statistically significant (log-rank *p* = 0.103). Abbreviations: DFS, disease-free survival; PNI, perineural invasion.

Disease-free survival was numerically less favorable in patients with perineural invasion. In this group, the median disease-free survival was 64.2 months (95% CI, 54.1–74.2 months), while the median was not reached among patients without perineural invasion. Nevertheless, Kaplan–Meier survival distributions did not differ significantly according to perineural invasion status (log-rank χ^2^ = 2.652, *p* = 0.103) (Figure 2).

## 4. Discussion

In this cohort restricted to patients with pathologic T3N0M0 colon cancer treated with curative-intent surgery, we investigated the clinicopathological characteristics associated with postoperative recurrence. Our main observation was that emergency surgery retained a significant association with recurrence after adjustment for other relevant clinicopathological factors. Although perineural invasion was associated with recurrence in the univariable analysis, this relationship was no longer statistically significant after multivariable adjustment, indicating that its prognostic contribution may overlap with that of other adverse clinicopathological features. Furthermore, the initially observed association between adjuvant chemotherapy and recurrence disappeared after multivariable adjustment, indicating that this relationship was largely attributable to confounding by indication rather than treatment inefficacy. Collectively, these findings suggest that emergency presentation captures prognostic information beyond conventional pathological risk factors and may improve postoperative risk stratification in patients with pathologic T3N0M0 colon cancer [18,19,20].

Stage II colon cancer remains one of the most heterogeneous clinical entities in colorectal oncology, with recurrence risk varying considerably despite identical TNM stage. Current international guidelines recommend considering several high-risk clinicopathological features, including T4 disease, lymphovascular invasion, perineural invasion, bowel obstruction or perforation, inadequate lymph node sampling, and poorly differentiated histology when making decisions regarding adjuvant chemotherapy. However, most published studies have evaluated these factors in mixed stage II populations, where the strong adverse prognostic impact of T4 disease may obscure the independent contribution of other variables. Consequently, the prognostic significance of individual risk factors, particularly emergency surgery, remains insufficiently defined among patients with pathologic T3N0 disease. By restricting our analysis to patients with pathologic T3N0M0 colon cancer, we minimized TNM stage-related heterogeneity and provided a more focused evaluation of clinicopathological variables associated with recurrence. This approach may explain why emergency surgery retained independent prognostic significance in our study, whereas several traditionally accepted high-risk features did not remain independently associated with recurrence after multivariable adjustment [18,19,21].

The biological mechanisms underlying the adverse prognostic impact of emergency surgery are likely multifactorial. In general, emergency colorectal cancer surgery may be required for acute tumor-related complications, and the associated inflammatory response, physiological stress, and increased postoperative morbidity may contribute to adverse oncological outcomes. These factors may facilitate circulating tumor cell dissemination, promote a pro-inflammatory tumor microenvironment, impair anti-tumor immune surveillance, and delay recovery, thereby reducing the opportunity for timely initiation of adjuvant chemotherapy. Furthermore, emergency procedures are often performed under suboptimal clinical conditions, where limited preoperative optimization and increased operative complexity may compromise the quality of oncological resection. Collectively, these mechanisms may explain why emergency surgery remained independently associated with recurrence even after adjustment for established pathological risk factors in our cohort. The relatively early separation of the Kaplan–Meier curves observed in our study may be consistent with the possibility that factors associated with emergency presentation and the perioperative period contribute to the subsequent risk of recurrence. However, this pattern should be interpreted cautiously, as the present study was not designed to determine the timing or mechanisms underlying the observed difference in DFS. Similar hypotheses have been proposed in recent clinical studies, which suggest that emergency presentation should be regarded not merely as a consequence of advanced disease but also as an independent determinant of adverse tumor biology and long-term oncological outcome [18,22,23].

Perineural invasion showed a significant relationship with recurrence in the univariable analysis, but this association was attenuated and became non-significant after adjustment for other variables. This result indicates that the prognostic information provided by PNI in pathologic T3N0 disease may not be fully independent of coexisting adverse clinicopathological characteristics. Although previous studies have linked PNI to unfavorable outcomes in stage II colorectal cancer, evidence supporting its ability to identify patients who are more likely to benefit from standard adjuvant chemotherapy remains limited. Baseline CEA concentrations were higher in patients who subsequently experienced recurrence in the between-group comparison; however, CEA was not significantly associated with recurrence in the univariable logistic regression analysis, suggesting limited independent prognostic value in this cohort. Furthermore, the apparent association between adjuvant chemotherapy and increased recurrence observed in the unadjusted analysis should be interpreted cautiously because patients receiving postoperative chemotherapy were more likely to harbor adverse pathological features. After accounting for confounding by indication, adjuvant chemotherapy was no longer independently associated with recurrence, emphasizing the importance of appropriate multivariable adjustment when evaluating treatment effects in retrospective observational studies [8,24,25,26].

The findings of the present study may have important implications for postoperative decision-making in patients with stage II colon cancer. Current international guidelines recommend considering multiple clinicopathological high-risk features when evaluating the potential benefit of adjuvant chemotherapy; however, the relative prognostic weight of these factors remains uncertain, particularly among patients with pathologic T3N0 disease. Our results suggest that emergency surgery identifies a subgroup of patients at substantially increased risk of recurrence independent of conventional pathological variables. Therefore, emergency presentation should not simply be regarded as a perioperative event but rather as an additional prognostic marker that may complement existing risk stratification models. Although our findings do not support the use of emergency surgery as a standalone indication for adjuvant chemotherapy, they suggest that these patients may benefit from closer postoperative surveillance, individualized multidisciplinary discussion, and incorporation of emerging prognostic biomarkers, such as circulating tumor DNA, into postoperative treatment planning. Future prospective studies are warranted to determine whether integrating emergency presentation with molecular biomarkers could further refine recurrence risk assessment and optimize adjuvant treatment strategies [15,27,28].

The present study has several notable strengths. To our knowledge, this is among the few studies specifically evaluating predictors of recurrence in a cohort restricted to patients with pathologic T3N0M0 colon cancer, thereby minimizing TNM stage-related heterogeneity that has limited the interpretation of many previous reports. Furthermore, the available clinicopathological data enabled multivariable analyses and a sensitivity model addressing confounding by indication, strengthening the robustness of our findings. The consistency between logistic regression and survival analyses further supports the reliability of the observed association between emergency surgery and recurrence. Despite these strengths, the findings should be considered in light of several limitations. The retrospective nature of this single-center study may have introduced selection bias and unmeasured confounding despite statistical adjustment. In addition, the modest cohort size and the limited number of recurrence events may have restricted our ability to identify associations of smaller magnitude. Importantly, the retrospective database did not systematically capture the specific indications for emergency surgery as mutually exclusive categories, the exact interval from symptom onset or hospital admission to surgery, or the use of preoperative decompressive procedures. In addition, tumor size and the extent of circumferential bowel-wall involvement were not consistently available as structured variables; therefore, residual confounding related to the clinical and pathological conditions underlying emergency presentation cannot be excluded. Another limitation was the lack of routinely available extended molecular data, including circulating tumor DNA, consensus molecular subtype classification, and other genomic biomarkers, which reflects the retrospective study setting [8,29]. Moreover, confirmation of these results in larger, prospective multicenter cohorts is necessary before their application to routine clinical decision-making.

## 5. Conclusions

In conclusion, emergency surgery emerged as an independent predictor of postoperative recurrence in patients with pathologic T3N0M0 colon cancer, even after adjustment for established clinicopathological risk factors. In contrast, the apparent association between adjuvant chemotherapy and recurrence was explained by confounding by indication and was no longer significant after multivariable adjustment. These findings suggest that emergency presentation provides prognostic information beyond conventional pathological variables and may improve postoperative risk stratification in patients with pathologic T3N0M0 colon cancer. Although emergency surgery alone should not be considered an indication for adjuvant chemotherapy, patients requiring emergency resection may benefit from closer surveillance and individualized multidisciplinary evaluation. Future prospective multicenter studies incorporating molecular biomarkers, particularly circulating tumor DNA, are warranted to validate these findings and further refine risk-adapted postoperative management strategies.

## Figures and Tables

**Table 1:** Baseline demographic, clinical, and pathological characteristics of the study population.

Variable	Category/Statistic	Overall (*n* = 118)
Age, years	Median (range)	64 (27–89)
Sex	Female	41 (34.7%)
	Male	77 (65.3%)
Comorbidity	Absent	53 (44.9%)
	Present	65 (55.1%)
Tumor location	Right colon	42 (35.6%)
	Left colon	76 (64.4%)
Type of surgery	Elective	88 (74.6%)
	Emergency	30 (25.4%)
Perforation	Absent	112 (94.9%)
	Present	6 (5.1%)
Number of examined lymph nodes	Adequate, ≥12	99 (83.9%)
	Inadequate, <12	19 (16.1%)
Perineural invasion	Absent	95 (80.5%)
	Present	23 (19.5%)
Lymphovascular invasion	Absent	87 (73.7%)
	Present	31 (26.3%)
Histological differentiation	Well differentiated	15 (12.7%)
	Moderately differentiated	86 (72.9%)
	Poorly differentiated	7 (5.9%)
	Unknown	10 (8.5%)
Microsatellite status	MSS	107 (90.7%)
	MSI	11 (9.3%)
CEA, ng/mL	Median (range)	2.30 (0.41–381.00)
CA 19-9, U/mL	Median (range)	10.15 (0.52–510.00)
Adjuvant treatment	No chemotherapy	40 (33.9%)
	FOLFOX/CAPOX	41 (34.7%)
	Capecitabine	37 (31.4%)
Adjuvant chemotherapy	No	40 (33.9%)
	Yes	78 (66.1%)
Recurrence	No	103 (87.3%)
	Yes	15 (12.7%)

Abbreviations: CA 19-9, carbohydrate antigen 19-9; CAPOX, capecitabine plus oxaliplatin; CEA, carcinoembryonic antigen; FOLFOX, leucovorin, fluorouracil, and oxaliplatin; MSI, microsatellite instability; MSS, microsatellite stable.

**Table 2:** Comparison of demographic, clinical, and pathological characteristics according to adjuvant chemotherapy use.

Variable	Category/Statistic	No Adjuvant Chemotherapy (*n* = 40)	Adjuvant Chemotherapy (*n* = 78)	*p* Value
Age, years	Mean ± SD	64.53 ± 10.37	62.00 ± 11.88	0.257
Sex	Female	11 (27.5%)	30 (38.5%)	0.237
	Male	29 (72.5%)	48 (61.5%)	
Comorbidity	Absent	15 (37.5%)	38 (48.7%)	0.246
	Present	25 (62.5%)	40 (51.3%)	
Tumor location	Right colon	18 (45.0%)	24 (30.8%)	0.126
	Left colon	22 (55.0%)	54 (69.2%)	
Type of surgery	Elective	39 (97.5%)	49 (62.8%)	<0.001
	Emergency	1 (2.5%)	29 (37.2%)	
Perforation	Absent	40 (100%)	72 (92.3%)	0.095 ^a^
	Present	0	6 (7.7%)	
Lymph node dissection	Adequate, ≥12	36 (90.0%)	63 (80.8%)	0.197
	Inadequate, <12	4 (10.0%)	15 (19.2%)	
Perineural invasion	Absent	40 (100%)	55 (70.5%)	<0.001 ^a^
	Present	0	23 (29.5%)	
Lymphovascular invasion	Absent	40 (100%)	47 (60.3%)	<0.001 ^a^
	Present	0	31 (39.7%)	
Histological differentiation	Well differentiated	8 (20.0%)	7 (9.0%)	0.403
	Moderately differentiated	27 (67.5%)	59 (75.6%)	
	Poorly differentiated	2 (5.0%)	5 (6.4%)	
	Unknown	3 (7.5%)	7 (9.0%)	
Microsatellite status	MSS	35 (87.5%)	72 (92.3%)	0.506
	MSI	5 (12.5%)	6 (7.7%)	
CEA, ng/mL	Median (range)	2.43 (0.45–381.00)	2.30 (0.41–143.00)	0.995
CA 19-9, U/mL	Median (range)	9.90 (2.00–510.00)	10.40 (0.52–298.00)	0.672
Recurrence	No	39 (97.5%)	64 (82.1%)	0.019 ^a^
	Yes	1 (2.5%)	14 (17.9%)	

Abbreviations: CA 19-9, carbohydrate antigen 19-9; CEA, carcinoembryonic antigen; MSI, microsatellite instability; MSS, microsatellite stable; SD, standard deviation. ^a^ Fisher’s exact test.

**Table 3:** Comparison of demographic, clinical, and pathological characteristics according to recurrence status.

Variable	Category/Statistic	No Recurrence (*n* = 103)	Recurrence (*n* = 15)	*p* Value
Age, years	Mean ± SD	62.57 ± 11.27	64.80 ± 12.56	0.493
Sex	Female	35 (34.0%)	6 (40.0%)	0.773 ^a^
	Male	68 (66.0%)	9 (60.0%)	
Comorbidity	Absent	46 (44.7%)	7 (46.7%)	1.000 ^a^
	Present	57 (55.3%)	8 (53.3%)	
Tumor location	Right colon	39 (37.9%)	3 (20.0%)	0.251 ^a^
	Left colon	64 (62.1%)	12 (80.0%)	
Type of surgery	Elective	82 (79.6%)	6 (40.0%)	0.003 ^a^
	Emergency	21 (20.4%)	9 (60.0%)	
Perforation	Absent	99 (96.1%)	13 (86.7%)	0.168 ^a^
	Present	4 (3.9%)	2 (13.3%)	
Lymph node dissection	Adequate, ≥12	88 (85.4%)	11 (73.3%)	0.260 ^a^
	Inadequate, <12	15 (14.6%)	4 (26.7%)	
Perineural invasion	Absent	86 (83.5%)	9 (60.0%)	0.073 ^a^
	Present	17 (16.5%)	6 (40.0%)	
Lymphovascular invasion	Absent	77 (74.8%)	10 (66.7%)	0.536 ^a^
	Present	26 (25.2%)	5 (33.3%)	
Histological differentiation	Well differentiated	14 (13.6%)	1 (6.7%)	0.804 ^b^
	Moderately differentiated	75 (72.8%)	11 (73.3%)	
	Poorly differentiated	6 (5.8%)	1 (6.7%)	
	Unknown	8 (7.8%)	2 (13.3%)	
Microsatellite status	MSS	94 (91.3%)	13 (86.7%)	0.630 ^a^
	MSI	9 (8.7%)	2 (13.3%)	
CEA, ng/mL	Median (range)	2.10 (0.41–381.00)	4.80 (1.58–143.00)	0.005
CA 19-9, U/mL	Median (range)	10.20 (0.52–510.00)	7.80 (1.00–171.00)	0.499
Adjuvant chemotherapy	No	39 (37.9%)	1 (6.7%)	0.019
	Yes	64 (62.1%)	14 (93.3%)	

Abbreviations: CA 19-9, carbohydrate antigen 19-9; CEA, carcinoembryonic antigen; MSI, microsatellite instability; MSS, microsatellite stable; SD, standard deviation. ^a^ Fisher’s exact test. ^b^ Exact test for multi-category variables.

**Table 4:** Univariable and multivariable logistic regression analyses of factors associated with recurrence.

Variable	Univariable OR	95% CI	*p* Value	Adjusted OR	95% CI	*p* Value
Male sex	0.772	0.254–2.345	0.648	—	—	—
Age, per 1-year increase	1.018	0.969–1.070	0.479	—	—	—
Presence of comorbidity	0.922	0.311–2.733	0.884	—	—	—
CEA, per 1-ng/mL increase	1.003	0.993–1.014	0.513	—	—	—
CA 19-9, per 1-U/mL increase	1.000	0.990–1.010	0.998	—	—	—
Left-sided tumor location	2.437	0.647–9.188	0.188	—	—	—
Emergency surgery	5.857	1.875–18.292	0.002	5.508	1.726–17.576	0.004
Adequate lymph node dissection, ≥12	0.469	0.132–1.665	0.242	—	—	—
Perineural invasion	3.373	1.061–10.723	0.039	3.021	0.886–10.300	0.077
Lymphovascular invasion	1.481	0.463–4.736	0.508	—	—	—
MSI	1.607	0.312–8.269	0.570	—	—	—
Adjuvant chemotherapy	8.531	1.079–67.437	0.042	Not included in the primary model	—	—

Note: Variables associated with recurrence at *p* < 0.10 in the univariable logistic regression analysis were entered into the multivariable model. Adjuvant chemotherapy was excluded from the primary model because treatment allocation was non-randomized and there was a potential for confounding by indication. Abbreviations: CA 19-9, carbohydrate antigen 19-9; CEA, carcinoembryonic antigen; CI, confidence interval; MSI, microsatellite instability; OR, odds ratio.

**Table 5:** Sensitivity multivariable logistic regression analysis including adjuvant chemotherapy.

Variable	Adjusted OR	95% CI	*p* Value
Adjuvant chemotherapy	3.515	0.377–32.768	0.270
Emergency surgery	4.079	1.207–13.779	0.024
Perineural invasion	2.324	0.662–8.161	0.188

Note: Adjuvant chemotherapy use was added to the primary multivariable model as a sensitivity analysis. Abbreviations: CI, confidence interval; OR, odds ratio.

## Data Availability

The datasets generated and/or analyzed during the current study are available from the corresponding author on reasonable request. The data are not publicly available because they contain information that could compromise the privacy of the participants.

## Abbreviations

AJCC	American Joint Committee on Cancer
ASA	American Society of Anesthesiologists
BMI	Body Mass Index
CA19-9	Carbohydrate Antigen 19-9
CAPOX	Capecitabine Plus Oxaliplatin
CCI	Charlson Comorbidity Index
CEA	Carcinoembryonic Antigen
CI	Confidence Interval
CRC	Colorectal Cancer
ctDNA	Circulating Tumor DNA
DFS	Disease-Free Survival
ECOG	Eastern Cooperative Oncology Group
ESMO	European Society for Medical Oncology
FOLFOX	Fluorouracil, Leucovorin, and Oxaliplatin
LVI	Lymphovascular Invasion
MSI	Microsatellite Instability
MSS	Microsatellite Stable
NCCN	National Comprehensive Cancer Network
OR	Odds Ratio
PNI	Perineural Invasion
SD	Standard Deviation
TNM	Tumor–Node–Metastasis
